# Robert Lee and his Undisciplined Medical Self: Life Writing, Character and ‘Technologies of Self’ in the Victorian Medical Profession

**DOI:** 10.1093/shm/hkae096

**Published:** 2025-01-22

**Authors:** James Bradley

**Affiliations:** History and Philosophy of Science Programme, School of Historical and Philosophical Studies, University of Melbourne, Melbourne, Victoria, Australia

**Keywords:** life writing, character, self, discovery, anatomy

## Abstract

Robert Lee was a divisive figure who, for much of his professional life, mismanaged his reputation. In this article, I use a diary written between 1837 and 1873 to explore the part character played in Lee’s fraught relationship with the medical profession. Special attention is paid to Lee’s dual use of life writing. On the one hand, he was an avid reader of biographies, memoirs and obituaries, which he then recorded in his diary. On the other, his diary writing was designed to audit his behaviour and then transform his self, making him a disciplined medical professional. Presented like this, his diary bears the hallmarks of a Foucauldian ‘technology of self’. However, Lee’s dual engagement with life writing revealed the character flaws that did much to damage his professional standing, and despite the diary’s use as a tool for self-fashioning, an unruly professional subject emerged from its pages.

## Prologue: Nil nisi bonum de mortuis?

On 1 March 1877, Sir James Paget gave his final presidential address to the Royal Medical and Chirurgical Society. Part of his task was to present ‘brief memoirs’ of those ‘important fellows’ who had died the previous year. Amongst a lengthy roll call, five obituaries were given ‘at great length, with more fulness of detail, analysis of characters, and feeling allusions to the usefulness of their life and works, and the lessons which they taught’.^1^ Mostly these were examples of diligence, brilliance and achievement. But one, that of Robert Lee, was less effusive. This was surprising for he had been a stalwart of the Society,

contributing twice as many papers to its Transactions as any living fellow. He had also had the honour of attracting the three largest meetings … which ever assembled in the room of the Society to discuss his papers upon the Use of the Speculum and upon Ovariotomy.^2^

Surely, this was a life worth celebrating? Apparently not—and to illustrate his point, Paget recalled some of the disputes that had engulfed Lee’s professional life, highlighting a specific problem with his character:

Lee, was a strong instance of that fault which sometimes goes with honesty; he was so sure that his convictions were the result of hard and well intentioned study, so sure that he sought the truth, and sought it in the right way, that he was wholly unable to believe that anyone with equal honesty and almost equal industry could arrive at conclusions different from his own.^3^

The notice ended with ‘a criticism on the character, value, and utility of his works in spite of his excessive opposition to all innovations’.^4^

This had not always been the received view of Lee and his achievements.^5^ To be sure, his reputation had been hard won. Born in 1793 near Melrose, Scotland, he studied at the University of Edinburgh graduating with an MD In 1814. He moved to London in 1817, aiming to establish himself as a physician with the patronage of Sir Gilbert Blane, who he had met while studying at Edinburgh. Blane used his influence to obtain a position for Lee in the household of the Honourable William Lamb (Lord Melbourne), caring for the future prime minister’s epileptic son. Subsequently, he studied in Paris (1821–22), then travelled the continent as a personal physician, before returning to London where he set himself up as a physician-accoucheur. After a protracted illness, he took up an appointment with Prince Vorontsov, governor of Crimea, hoping the climate would improve his health. In 1826, he returned to London much improved and once again set about fulfilling his ambitions. He was a restless figure. In 1834, he was appointed Regius Professor of Midwifery at the University of Glasgow (probably through the patronage of Lord Melbourne) but resigned soon after his inaugural lecture, quickly returning to London. From the 1830s until the end of the 1840s, his time was consumed by teaching, practice, anatomical discovery and, most importantly, controversy: a series of disputes with the Royal Society became a defining feature of his life.^6^

The controversy will be dealt with in more detail below, although here it is worth mentioning that it was built upon his twin interests in anatomy and obstetrics. In the late 1830s, he believed he had discovered a separate nervous system connected to the gravid uterus that overturned received anatomical wisdom. In the years that followed, the discovery was disputed, accepted and then rejected in a process that revealed corruption at the heart of the Royal Society. His ultimate triumph made him an unlikely figurehead for the reform of science during the 1850s and he was honoured by a lengthy puff-piece in the *Lancet* celebrating his political triumphs and anatomical discoveries.^7^ Shortly after, the Royal College of Physicians rewarded him with the Lumleian (1856–57) and Croonian Lectureships (1862), followed by the ultimate accolade of the Harveian Oration (1864). His newfound reputation had also given him a platform to campaign against many of the period’s obstetric and gynaecological innovations: anaesthesia in childbirth; the use of the speculum; and ovariotomy.^8^ The ‘three largest meetings’ alluded to by Paget revealed considerable support for his agenda.^9^

Paget was, however, more interested in the present day, and Lee’s achievements were decidedly in the past. By the late 1870s, his conservative obstetrics had been surpassed by advances that made him look old fashioned.^10^ Moreover, what had appeared in 1850 as a major innovation in nervous anatomy was, in 1877, anything but—the *Lancet*’s 1851 prediction that his fame would rest upon the discovery of the uterine ganglionic nervous system (‘one of the most important ever made in human anatomy’^11^) wide of the mark. Worse still, Paget implied that Lee was an undisciplined professional on two counts: opposing ‘*all* innovations’ (my emphasis) was a recipe for stasis and, therefore, fundamentally *against* science; failing to see merit in an opponent’s views or recognise their ‘honesty’ and ‘industry’ was distinctly uncollegial.^12^

Notably, the *British Medical Journal* had already reached its own verdict on Lee’s life: he was ‘a bitter conservative’, adding the vices of impetuosity and prejudice to Paget’s list of moral failings.^13^ The *Lancet* had forgotten its earlier effusiveness, merely providing a brief, factual death notice where his discoveries were barely mentioned and he was simply presented as ‘the author of numerous works in the department of medicine with which he had associated himself’.^14^ Only the *Medical Times and Gazett*e *saw his life as worth celebrating.*^15^ It, at least, said nothing but good of the dead.

## Character, Identity and Self in Robert Lee’s Diary: An Introduction

Of the four obituaries cited, two were from the most widely read British medical journals; publications that policed the social and scientific boundaries of the ‘imagined community’ of the medical profession.^16^ Lee’s science, from this perspective, was far removed from the cutting-edge of the laboratory and operating theatre. His professional conduct, however, had always been questionable, so while he would not have accepted the posthumous verdicts on either his discoveries or his conservative obstetrics and gynaecology, the archives show he was fully aware of his divisive personality and reveal the lengths he went to curb it.^17^ Indeed, a diary he kept sporadically from 1837 until a few years before his death bears witness to this, charting his troubled progress through the upper echelons of medical London.^18^ On the surface, he was a successful medical man, juggling the roles expected of a member of the medical élite in reform-era London: hospital and teaching appointments; clinical and anatomical research; and private consultations that supported a large extended family. But the diary paints a different picture of a cruel and morally bankrupt profession, where financial gain was often achieved at the expense of scientific investigation and where greed triumphed over duty. Underlying this was a measure of personal bitterness and entitlement; private signifiers of an unprofessional medical self that all too often appeared in public settings, doing much to undermine Lee’s professional credibility.

There are many sources which could have been used to explore the interaction between the public and private faces of Lee’s persona. His disputes were often conducted in the glare of the medical press or the pages of his textbooks. Traces of his fractious nature can be found too in the correspondence of his antagonists.^19^ Undoubtedly, a deeper engagement with these sources would provide insights into the evolution of the medical profession, including the development of its boundaries and contours. Here, however, I narrow my focus to the relationship between life writing and the self. Not only does the diary lend itself to this approach, but critical analysis of its contents allows significant theoretical insights into the moral function of life writing and the part this set of practices played in schooling character and identity.

Lee himself engaged with life writing in two ways. On the one hand, he was an avid reader of biographies, memoirs and obituaries, which he selectively recorded in his diary. On the other, his diary-writing was designed to audit his behaviour and then transform his self, making him a disciplined medical professional. Presented like this, his diary bears the hallmarks of a Foucauldian ‘technology of self’, which aimed to ‘transform’ the self to ‘attain a certain state of perfection, happiness, purity, supernatural power’.^20^ Technologies of self operate upon the subject’s ‘body’, ‘soul’, and ‘thoughts’ and were tools that, in dialogue with particular dispositions and identities, shaped and reshaped the self.^21^ However, Lee’s dual engagement with life writing not only illustrated the disciplinary intention of the practice but also laid bare the character flaws that would do much to damage his professional standing: despite the diary’s use as a tool for self-fashioning, an unruly professional subject emerged from its pages.

In working through this argument, I will highlight the role that character played in self-fashioning. The word ‘character’ abounds with equivocation. Putting to one side its use in literary contexts, it sometimes describes surface appearances, while at others the public reputation of an individual and at still others a mechanism that controls behaviour.^22^ My analysis of Lee and his reputation uses the third of these definitions. In his world, a well-formed character disciplined the individual’s ‘will’ in such a way that it performed virtuous acts (e.g. ‘disinterestedness’), while resisting those controlling passions that impelled individuals towards vice (e.g. ‘vanity’). Character mediated between the moral frameworks that were part of the cultural fabric and the individual’s self-understanding of who they were and what they stood for. Ideally, it would become part of the *habitus*, so ingrained that decisions would be intuitively made and then unconsciously carried out.^23^

Character is, of course, an abstraction. However, it gets close to how Lee and his peers understood themselves. Rarely a subject for medical historians of identity,^24^ it was fundamental to nineteenth-century conceptions of morality and conduct.^25^ Bearing a good character, outwardly exhibiting the marks of professional virtue, was the bedrock upon which a good medical reputation was built: here behaviour was understood as a symptom of an individual’s moral core. As the obituaries indicated, and as will be explored further, Lee did not bear a good character; his diary provides plenty of evidence that he was aware of this and wished to change it by transforming his inner self and modifying his outward behaviour.

My engagement with Lee’s diary will touch upon aspects of medical professional life and identity that were features of his time. However, in what follows, medical identity *per se* sits quietly in the background: I never describe a fully-formed professional identity that was the end-goal for Lee’s self-fashioning. He certainly had a vision of what he wanted to be: a successful practitioner diligently serving the medical needs of London’s women, both rich and poor; a respected teacher of his craft in St George’s Hospital Medical School; and a front-rank anatomist recognised for major discoveries. But medical identities during this period were in flux.^26^ Rather like Humphry Davy trying to forge a scientific self from a culture that had not yet produced the identity of ‘scientist’, Lee faced a patch-work of evolving identities from which to make his medical self.^27^ To be sure, the identity of anatomist was well-defined and later we will see Lee attempting to fashion himself in the image of William Hunter. He would also draw off older identities, including that of physician.^28^ The identity connected to obstetrics and gynaecology, however, was as emergent as the specialism itself. Lee, himself, would try and fail to shape this identity in line with his conservative beliefs by producing identity-defining histories.^29^

A further reason for pushing professional identity into the background is its historiographical overdetermination. Historians, like Brown and Moulds, have been adept at identifying the overarching frameworks that shaped medical selves. But taking what are, in effect, identity templates and then mapping them to Lee’s diary creates analytical problems. This is mainly due to the capacious materials from which selves were made; Lee’s self-fashioning drew upon a mix of history, theology, politics, law and literature, as well as medical culture itself. Indeed, the virtues that were at the heart of medical identity were shared with wider society and were part of a larger moral framework that transcended medical identity. To illustrate, Stana Nenadic identified the virtues of the ‘ideal doctor’:

affectionate, moderate, temperate and industrious, he achieved distinction through his own endeavours, was simple in his pleasures and regular in his habits. Cheerful sociability was stressed, as was benevolence.…Plainness not only of manner but also of dress came be viewed as essential.^30^

None of these were categorically medical virtues; all were expected of respectable middle-class men in early Victorian society and were not only attributes of manliness, but were also preached to congregations of every stamp.^31^

## Diaries, Commonplace Books and the Power of Life Writing

In my reading of Lee’s diary, I was drawn to the complex interplay of different forms of life writing. Technically, however, it was not a diary at all. The book in its pristine state, as yet uncovered in his frantic scrawl and the slippages in and out of shorthand, would have been owned by many of his contemporaries. Its title, *The Literary Diary or Improved Common-Place-Book*, pointed towards its origins and uses.^32^ A commonplace book was designed for recording reading; where useful passages could be copied into the book, sometimes with added commentary. What was useful to the reader was, of course, an individual matter, but often it was related to virtue. Historians have noted that the eighteenth century was the high-water mark for commonplace books, although more recently it has become clear that they remained popular through the Victorian era and beyond.^33^

Owners of Lee’s commonplace book were encouraged to use an indexing method created by John Locke, disclosing the connections between commonplacing and Locke’s educational philosophy. According to the printed introduction, commonplace books acted as ‘promptuaries or storehouses, wherein to reposit our own ideas, as well as the most valuable thoughts of others, to be ready at hand when wanted’.^34^ The book would thus become a warehouse of useful knowledge, discovered and recorded by its author; a place where biographies, obituary, story or verse might provide vital life lessons—knowledge to forge future conduct. It was nothing less than a tool for transforming the *tabula rasa* into a fully-formed and cultured self.

There was a thin line between the function of a diary and that of a commonplace book, although some have argued for a distinction between inward-facing diary writing and outward-facing commonplacing; the former focussing upon the author’s interiority, while the latter connected the author, through their reading, to the wider world.^35^ Nevertheless, while diaries might appear to give more immediate access to the diarist’s thoughts and feelings, commonplace books also recorded lived and felt experiences, albeit mediated through cultural consumption and reproduction. Nevertheless, both fulfilled the role of ‘technology of self’.^36^

**Fig. 1.**
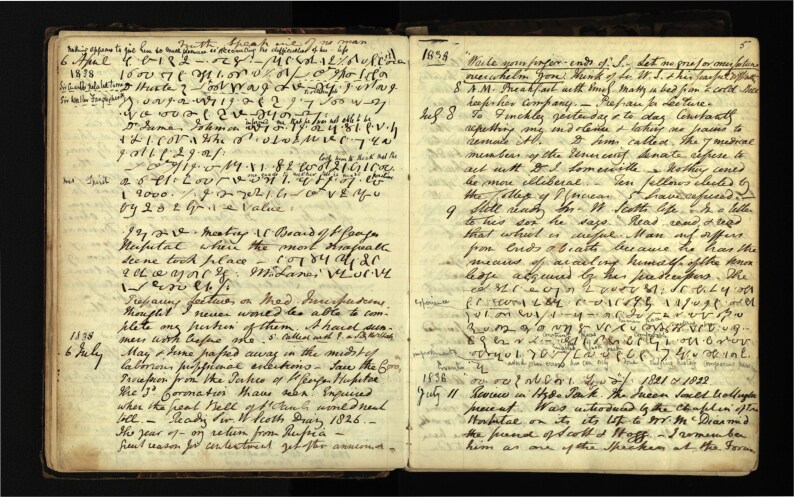
Page from Diary of Robert Lee, 1837–1877, Wellcome Collections, MS 3281. Note the use of shorthand, with an attempt at translation by Lee’s son. Many events are recorded here including the coronation of Queen Victoria and the internal politics of London University. He also records that he is ‘Reading Sir W. Scott’s Diary, 1826’ and quotes from it

While a properly compiled commonplace book relied upon an effectively organised index and the creation of discipline-based topics (arts, mathematics, natural philosophy, *etc*. were suggested), Lee used his in a hybrid fashion.^37^ On one level, it was nothing more than a chronologically ordered diary, with additional remarks sometimes added days, weeks and even decades later. The index itself remained largely unused, although it and the blank pages at the front and back were covered in dates, page numbers and comments, many of which were added posthumously by one of his sons. On another level, in keeping with the spirit of the commonplace book, it presents a complex interplay between autobiographical observation and the ethical force of life writing (See Figure 1).

Commonplacing and diary writing were themselves both forms of life writing. At the same time, as a practice, it encouraged the reproduction of other forms of life writing, particularly biographies, memoirs and obituaries. This interaction is hardly surprising. One of the fundamental roles of life writing was instruction—to teach life lessons and, in an ideal world, produce particular types of ethical subject.^38^ In the context of the social history of medicine, Stana Nenadic’s ground-breaking meta-biography of Matthew Baillie illustrated the normative functions of medical biography in the early nineteenth century and how it participated in forming medical selves. As the medical press expanded its scope, obituaries became as important as biography for eulogising exemplary men and presenting exemplary versions of medical identity, with the assumption that these ideals would have a formative role in medical self-fashioning.^39^

This was certainly one of the ways that Lee mobilised life writing. In particular, it allowed him to explore aspects of his professional life that vexed him, including the perceived lack of recognition he received for his scientific investigations. In an ideal world, a diligent man of science and medicine like Lee would have been rewarded for his discoveries with accolades, medals and an increased private practice. This was a trajectory identified by both M. Jeanne Peterson and Anne Digby,^40^ and it remained one of the most persistent concerns of Lee’s life, reflected in his reading and recording of the lives of others, particularly fellow medical practitioners. With the notable exceptions of Marshall Hall and William Hunter, the latter revealed as Lee’s principal inspiration, most of his medical subjects failed to achieve this balance, particularly Charles Bell, whose life Lee returned to several times.

Like Marshal Hall, Lee was involved in many disputes—several of which played out in the medical press and were driven by his conservative view of treatment.^41^ But the dispute to which he invariably returned was the Royal Society and its rejection of his nervous discoveries. It was here that his inability to live up to his biographical subjects was not only most glaringly obvious but also revealed the failure of the diary’s disciplinary purpose. Indeed, Lee was never able to fashion himself into the ideal man of science and medicine. For this he partly blamed the profession’s priorities; but like his critics, he also identified his own character as a problem—especially his inability to bend his will to the behavioural requirements of his profession. Nowhere was this more seen than in Lee’s disputes with the Royal Society that lasted for much of the 1840s.

## Robert Lee Versus the Royal Society

The disputes that did so much to define Lee’s life have been briefly described by Diana Manuel.^42^ Nevertheless, we must revisit this episode for the light it throws upon how Lee’s troubled relationships with his peers, as well as providing vital context for many of his commonplace reflections. In the late 1830s, Lee made a discovery which he believed overturned anatomical knowledge; specifically, that there was a separate ganglionic nervous system that supplied the gravid uterus. The discovery was made using traditional anatomical techniques, with only limited use of comparative anatomy and microscopy. He relied upon a supply of women’s corpses at different stages of pregnancy, a privilege granted by his lecturing position at St George’s Hospital. As pregnancy progressed, he identified significant changes to the size of the ganglia, which, he speculated, provided added nervous power to the pregnant uterus.

Much of the dispute revolved around the reality or not of the nervous ganglia. Here, Lee relied upon preparations and illustrations to persuade people of his discoveries. In these, his supporters saw the evidence of ganglia, while his opponents argued that they were merely connective tissues. Initially, the publication of his first paper on the subject was rejected by the Royal Society for exactly this reason. Indeed, the members of the Physiological Committee, reviewing and refereeing for *Philosophical Transactions*, were divided on whether his discovery was a discovery at all. Until just before his death Astley Cooper, at the time *de facto* chair of the committee, remained unconvinced—although he, alongside Richard Owen, had a change of heart.^43^ Ultimately, the discovery was accepted by the committee, and the paper was published in *Philosophical Transactions*.^44^ For a while, the matter appeared settled.

But the discovery was again challenged in 1845 when the Council of the Royal Society awarded that year’s Royal Medal for Anatomy and Physiology to Thomas Snow Beck on the recommendation of the Physiology committee. At the time of the committee’s recommendation, Snow Beck was ineligible for the award, which was reserved for the most important anatomical or physiological discovery published in Europe during the previous year. No such paper had been written, read or published; indeed, no public announcement of findings had been made whatsoever. When it finally saw the light of day, a considerable time after the committee’s recommendation, the discovery could hardly be described as a discovery at all. Rather, it was a refutation of Lee’s nervous ganglia using specimens Lee had lent the younger anatomist. Lee was furious and martialled a challenge against the society’s ruling faction. The matter rumbled on for several years until eventually, after a torrid special general meeting, secretary Peter M. Roget was forced to resign following revelations of procedural irregularities and underhand behaviour by both the Physiological Committee and the Council. The Earl of Northampton, the president, resigned soon after ushering in a period of reform.^45^ As we have seen, the influence Lee gained from his victory had waned by the mid-1860s, and by his death, his reputation was a shadow of its former self.

## Lee and the Uses of Life Writing

On the face of it, the fury over Lee’s discoveries is a conundrum.^46^ The internecine politics of London undoubtedly played their part, although there is no obvious ideological reason for resistance to Lee’s discovery—the challenge to former authority was unthreatening, God remained firmly in his heaven and women were maintained in their subordinate role, despite their developmentally superior nervous systems. There were, however, suspicions that having found nervous ganglia supplying the uterus, he then found them everywhere he looked,^47^ amplifying previously identified character flaws that may have done much to undermine his credibility. But before we explore Lee’s undisciplined medical self, we need to examine his reading and the way this was used to articulate his feelings when faced with triumph or adversity.

Lee was an avid reader of biography and, where it involved someone of significance, an assiduous recorder of obituaries. Reading his diary against the events of his life shows the extent to which he framed his experience through biographical and related forms of life writing. Aside from those dealt with in this article, these included: John Hunter’s son; Astley Cooper; the prophet Mohammed; Lord Robert Clive; Justice Story; Guillaume Dupuytren; Lord Chief Justice Tindal; the reverends Thomas Chalmers and George Whitefield; and Joseph Priestley. Each one spoke to specific concerns he had about the inter-relationship of character, profession and discovery. That some of those memorialised were lawyers is unsurprising; both medicine and law combined precarity with huge personal demands.^48^

A typical pattern of Lee’s reading occurred in March 1841, during his attempts to persuade the medical establishment of the reality of the nerves of the gravid uterus. He was ‘disgusted with medicine and did nothing’.^49^ Doing nothing did not preclude pertinent reading: in this case, the memoir of the lawyer and politician Samuel Romilly, who frenziedly killed himself after his wife’s sudden death. The professional struggles described by Romilly echoed Lee’s experiences:

I have nothing but hours before me I can’t look any way without seeing Barristers or Attorneys. This is another sacrifice which I have made to the profession which nothing but inevitable necessity forces me to submit to. Which I every day feel more & more that I am unfit for, & which I dislike the more, the more I meet with success in it.^50^

To this, he added the terse note: ‘cut his throat at 61’. Lee had a choice in what he recorded. He might, in this instance, have chosen passages outlining Romilly’s career as an outstanding humanitarian advocating against slavery and for judicial and penal reform. But instead, he homes in on the burden of a professional vocation. By connecting Romilly’s suicide to professional struggle, Lee appears to agree with those contemporary observers who felt that Romilly’s professional life was an important factor in his unstable mental state, while indexing his own concerns about his place in the medical profession.^51^

Days later, after reading David Brewster’s *Martyrs of Science*, he would reflect upon the fate of those who made great discoveries. Brewster’s verdict on Galileo struck a resonant chord—‘all his work met the most violent of opposition’—a situation Lee knew only too well, while no doubt allowing him to imagine his own high place within the pantheon of discovery.^52^ Later on, I will explore his dealings with Sir Walter Scott’s *Memoir*, but often he was drawn to the obituaries of fellow medical men, like Charles Bell. Strikingly, whether it was Romilly or Scott, Galileo or Bell, for Lee the moral of the story was the same: the price of self-sacrifice, dedication and discovery was disappointment, opposition or neglect. But he also recognised that character played a part. This was evident in those cases where he had personal knowledge of the individual—his commonplace reading was bolstered by marginalia, which sometimes contradicted the original source.

Lee’s recording of John Sims’s obituary provides an excellent example, combining a copy of the *Morning Chronicle*’s sombre account of Sims’s life with supplemental observations made by himself and other acquaintances. Lee copied out the paper’s verdict that Sims was an ideal medical professional who had died in the line of duty due to ‘a malignant fever, of a low typhoid character, which he is supposed to have caught at the St. Marylebone Infirmary’. The *Chronical* highlighted his ‘skill and disinterested kindness’ including the ‘gratuitous’ treatment of poor patients.^53^ Superficially, he was the living embodiment of a good medical man: dedication to science, self-sacrifice, moral probity and kindness were bound together by the virtue of disinterestedness. But Lee’s acquaintance with Sims (he had known him from his student days in Edinburgh) allowed him to present a more nuanced and less heroic picture. ‘In the latter years of his life he became jealous and dissatisfied with everything’, he scribbled.^54^ The next day, the pharmacist Jacob Bell, who had also been an intimate of Sims, visited and informed Lee that

Dr Sims had often expressed his dissatisfaction with the Medical Profession—He said the vexation, want of leisure—misunderstandings & want of confidence between medical men had given him far greater pain than pleasure from his Profession.^55^

While this was an accurate reflection of Lee’s own view of the costs of practising medicine, there was more. The obituary had failed to describe Sims’s involvement with London University and the disgust he felt at the politicking that was endemic there. Institutional politics entailed conflict as a matter of course: ‘all who embark in Politics as Dr Sims did must lay their account with the opposition & every kind of personal annoyance’. This led Lee to counsel himself with advice that would be ignored more often than not: ‘avoid giving offence whenever you possibly can’.^56^

Sims’s death occurred before the major struggle of Lee’s life, but around the time he transcribed the obituary, his commonplace book was soaked in professional disappointment, jealousy and conflict—the day-to-day snubs from knighted neighbours, the incompetency of senior colleagues, the constant grind of getting a living and achieving approbation and the insecurity of his own expertise—all these permeate his writing.^57^ He could see how easy it was to become stuck in the swamp of medical politics. And yet, the constant hankering for fame and posthumous reputation would dump him in the mire time and again, with insecurity and bitterness the results. These effects were reflected in his several readings of the life of Charles Bell.

## Discovery and Disappointment: Reading the Life of Charles Bell

For much of the period before 1850, Lee was embroiled in the dispute over his discovery of the uterine nerves, trying to establish that he had discovered something of note. In the early 1840s, he was making headway: publication in the *Philosophical Transactions* was followed by a term on the Council of the Royal Society (1843–44).^58^ When he sat down, in the spring of 1843, to read the *Quarterly Review*’s obituary of Charles Bell, he appeared secure in his newfound status as a front-rank anatomical discoverer and was more concerned with other aspects of his professional life, particularly the relative scarcity of paying patients—a problem Bell also faced.^59^ It would not be the last time that Bell’s life would become a lens for his own. He returned to the subject 2 months later for similar purposes before revisiting Bell’s life in 1846.^60^ By then, his focus had changed. He was less concerned with practice and more with protecting his status as a trustworthy scientific investigator.

The initial obituary, published in the *Quarterly Review*, was masked as a review of several of Bell’s works, as well as his brother-in-law’s defence of his priority in the spinal and facial nerves (1839).^61^ In reality, this was the first posthumous biography of Bell, running to over 23,000 words spread across 40 pages of the Tory journal. Written anonymously by the Scottish-born physician Robert Ferguson, it gave an unprecedented view of Bell’s life drawn from personal correspondence, much of which would later appear in redacted form in Bell’s *Letters*.^62^ It was a vindication of Bell, his discoveries and his character, and was written by a man well acquainted with him.

Despite a ‘warm friendship with Sir Charles Bell’,^63^ the deeply personal nature of the biography would have been news to Lee, even if the details of Bell’s battles for priority were not. Indeed, based mainly upon the private correspondence between Bell and one of his elder brother, the lawyer George Joseph, the inner turmoil of Bell’s life would have been news to almost everyone, including, perhaps, Bell himself. The review has a claim to being the most important piece of life writing about Bell, providing a foundation for subsequent biographies, including Amedée Pichot’s full-length work of 1860.^64^

Ferguson presented Bell as a kind and compassionate individual possessed of humour and good grace—if at times ‘out-spoken’ and ‘unworldly’. But more than that, it developed the theme of Bell as a romantic scientific hero, thwarted by forces that sought to deny him the glory that were the due for his discoveries. In later life, tired of the conflict of London, he returned to his native Edinburgh, seeking to recapture the joys of his youth, only to have his hopes dashed by dead friends, and an ever-diminishing practice and lecture roll. His dedication to science and his disinterestedness meant he never commanded a large and remunerative practice, sacrificing medical fortune for the glories of anatomical and physiological discovery.

Much of this accorded with Lee’s self-perception. While he copied out parts of the private correspondence that reflected Bell’s enormous self-belief, he also emphasised the sacrifices Bell had made in making his discoveries, including the income lost from not having time to consult. Lee briefly noted the attempts that were made to ‘wrest’ the credit for Bell’s discoveries from him. But he concluded by copying the following passage about Bell’s return to Edinburgh: ‘he seemed to walk in a city of tombs … – found no one to sympathise with him or visit old places – often felt it as a dream’, followed by Bell’s judgement on the failures of those contemporaries who had abjectly failed to change their behaviour in the light of their character—the ‘one trait on which all depends’.^65^ Finally, he noted the rapid decline in the size of Bell’s class and his dwindling financial circumstances.^66^ In all, Lee had reduced Bell’s life from tens of thousands of words to a few hundred, incorporating what were, for him, the salient facts.

Lee was highly selective in his choice of passages from the *Review*, failing, for example, to note Bell’s scientific renown amongst the great and the good of Europe.^67^ But in the late spring of 1843, relatively secure in his own discovery, he was more concerned about his day-to-day practice—he never seemed to have enough patients and it was extraordinarily costly supporting two households, one in London and the other in Brighton, and his experimental endeavours.^68^ His emphasis upon Bell’s professional failure neatly summarised his current fears. But there remained other elements that chimed with the function of Lee’s commonplace book. On the one hand, the narrative, as reshaped by Lee, provided a stark reminder about the fleeting nature of success—no matter the importance of Bell’s discoveries, he still died impoverished. On the other, given that only a few lines before Lee had scrawled two derogatory judgements on Bell’s character—‘no practical tact’ and ‘extravagance the cause of all this’—his summary cautions the need to maintain probity in the face of life’s travails, while the ‘one trait’ provides a stark warning about character, its malleability and its necessity for professional success.

It was hardly coincidental that three entries later, in July, his attention returned to Bell’s life. Once again, he revealed that he was more interested in the diminished financial returns of discovery than challenges to a discovery’s originality. Perhaps his reading of Ferguson’s obituary had recalled to him a *Medical Gazette* editorial, written in 1838. Here, the editor repeated comments made by Bell about the adverse impact discovery had upon private practice.^69^ Lee laboriously copied out the relevant passage in shorthand, including Bell’s belief that ‘the philosophy & the practice of the profession’ were ‘necessary to each other’, but that ‘the prejudice of society’ ran ‘against this association of objects, so natural & so just’. For ‘on each occasion’ Bell’s name had been joined with science, it had been necessary for him ‘to double’ his ‘attention to practice’.^70^ Indeed, the editorial concluded, that the ‘most successful practitioners … [were] those who devote only a small portion of time to anatomy or physiology or any abstract scientific investigation’ because they understood that ‘a knowledge of the art of medicine or surgery’ was the only thing that counted with potential patients.^71^ Furthermore, not only might scientific discovery fail to translate into riches, it also made the discoverer vulnerable either to contest or derision. ‘If he should make what … he would call a fortunate discovery’, the writer pointed out and Lee duly recorded, ‘he has to run the gauntlet of numerous competitors, for the honor [*sic*] of originality and pirate or not, he must fight, or give up his claim’.^72^ Here Lee’s transcription ended, neglecting to address the context of the *Gazette*’s comments: the priority dispute that was being waged between Marshall Hall and Richard Grant, on the one hand, and George Newport on the other.^73^ Thus, he concluded: ‘resolved to devote myself earnestly to practice, to abandon all pursuits which could make the public suppose as Sir. C. Bell said that I am anything but a practitioner’.

Lee returned to Bell’s life in August 1846, at the height of his dispute with the Royal Society. This time the focus was solely upon the recognition of discovery and priority. By now the dispute had all but destroyed his credibility—‘I was held by all to be a convicted blunderer in Anatomy … the greatest that had ever appeared’. And while his private practice was affected, his main concern was the ‘destruction’ of the system that he believed would guarantee his posthumous reputation.^74^ His attention fell upon a recently published article from *Chambers’s Edinburgh Journal*,^75^ which outlined the scale and nature of the challenges faced by Bell.^76^ Rather like Lee, Bell had had to fight against ‘opponents … arrogant and clamorous in their assertions’. Ultimately truth prevailed, a narrative that probably allowed Lee to steel himself for the fight ahead, with Bell’s life reminding him of the sacrifices required of great scientists, the jealousies that arose from pigmy opponents, before concluding, using a direct quote, that the only rewards available to the disinterested man of science were ‘the pleasure arising from the pursuit of natural knowledge’ and ‘the society’ of other ‘men of Science’.^77^

## Robert Lee’s Bad Language: Writing the Wrongs of the Past

Bell’s life provided many lessons for Lee, not least the need to guard an always threatened legacy. For all of Ferguson’s efforts to portray Bell as a martyr to science, there remained little doubt in his mind that Bell would be remembered as a great discoverer and a supreme anatomist and physiologist. And despite the challenges to his priority, Bell’s name was still associated with the motor and sensory nerves—he remained in the pantheon of discovery, if not sitting next to William Harvey then somewhere close by. Lee was convinced that his own nervous discoveries were no less momentous, and, as we have seen, the *Lancet*, amongst others, agreed (or at least it did in 1851).

However, by the end of the 1850s, his confidence in his reputation had all but dissipated and the older he got, the more he was driven to find out who had been responsible for the Royal Society affair. Thus, in 1864, he was ‘greatly depressed in spirits, & feel my strength and mental powers gradually becoming exhausted’. Recognising the effects of ageing and that his life was nearing its end, he saw only ‘Hollowness and heartlessness all around’.^78^ But the following year, he was spurred into action by the posthumous publication of Benjamin Brodie’s *Autobiography*.^79^ Brodie had been chair of the Physiology Committee when the Royal Medal had been awarded to Snow Beck, and he had harboured a persistent belief that Brodie was responsible for his troubles, leading to several attempts at excavating Brodie’s true character.^80^ He ‘had a long interview with Mr Lawrence’ where he asked ‘if he had seen the biography of Sir B. Brodie’. Lawrence had not, but also ‘had no desire to see it’.^81^ About the same time, he had written to Alexander Shaw, Charles Bell’s brother-in-law. Shaw’s reply implied that Lee had been asking about Brodie’s dealings with Bell and he refused to be drawn on the subject.^82^

But Lee was constitutionally unable to let matters lie. In the spring of 1866, we find an intemperate series of entries where he imagines writing a history of the controversy. This would involve listing the ‘names of all with whom I came into contact and collision’. ‘Every detail, however minute’ was to be recorded, ‘nothing here after will be unimportant’. Writing this history was critical to Lee’s future posterity. ‘It is necessary that a just estimate should be formed of the talents and characters’, he said with a degree of measured calmness before parenthetically ranting: ‘their characters & talents have already been justly estimated – these names unworthy of notice’. Seething with the injustice, his history would finally set wrongs to right.

If it had been written, it would have been a grandiose affair, encompassing the entire state of anatomy and physiology in Europe at the time of the controversy. But above all, it would lay bare the ‘conceit & ignorant condition of the Fellows of the Royal Society. The professors of Anatomy & Physiology their ignorance & perversity’. He then listed the culprits, many of whom had been on the committee: William Bowman, Richard Owen, Francis Kiernan, Richard Partridge, William Lawrence, Peter Roget and William Sharpey. ‘The truth is that none of these men possess any scientific reputation’, he added.^83^ A few months later, he would revise his list when he once again considered writing his history. Angrily striking out the names of those who had died, the roll call of infamy poured out: ‘Sir B. Brodie Kiernan Todd Bowman Owen Sharpey & Swan Richard Quain’. And then in a final judgement that was as intemperate as it was inaccurate, he concluded ‘None of them ever made a dissection’.^84^

The history remained unwritten. And neither would it appear in any of his son’s fitful but failed attempts at writing his father’s biography.^85^ While there can be little doubt that a wrong was done to Lee, his expression was intemperate, deeply emotional and bordered upon the irrational. Indeed, his emotions often got the better of him. For example, just as the Royal Society affair was nearing its conclusion and despite the huge toll of fighting his opponents, he still found time for a furious dispute with James Young Simpson over the treatment of *placenta praevia*.^86^ The tone of the dispute was so rancorous that one commentator criticised both Lee and Simpson for the ‘great vexation and … humiliation’ brought to the profession.^87^ And, only 3 years before, he had had a deeply unpleasant exchange with Robert Paterson over the *corpus luteum*.^88^

Lee, himself, knew how easily he was drawn into controversy and cautioned himself to avoid it. As he said early in the journal, ‘Resolved … [t]o disregard the petty disputes & cabals & to hold strictly to the scientific part of my profession’.^89^ But, as his proposed history demonstrated, it was a resolution made in vain. All too often he failed to curb his untoward passions despite being acutely aware of the problem from an early point.

## James Clark, Walter Scott and the Failed Management of the Professional Self

Nowhere, perhaps, was Lee’s failure to manage his professional self more apparent than in his reflections on Sir James Clark, which were framed by his reading of the life of Sir Walter Scott. Sometime around the middle of 1838, Lee conducted a routine audit of his life and career. Recalling his move to Golden Square, Soho, some 8 years earlier, he reflected that ‘[m]y footing here I still consider extremely insecure’. ‘I am’, he continued, ‘disliked by many of my professional brethren’.^90^ In the very next entry, this observation is amplified when a visiting friend mentions the ‘brilliant success of Sir James Clark’. Clark, Lee noted, possessed an ‘immense practice … while my house is almost deserted’, the result of being ‘prudent in the highest degree’, a man who ‘said nothing of his own affairs, & took care to offend none’. There were lessons to be learned. ‘Is it still too late to retrieve—to become mild & respectful to all—to recover that which I have imprudently sacrificed’, Lee wondered in the light of Clark’s success.^91^

Clark provided a benchmark for Lee’s progress. Five years his senior, Clark had risen from a humble background to become a court physician, by way of the Royal Navy and the University of Edinburgh. His MD dissertation on the effects of cold on the human body set his future path; he would focus on the impact of climate on the sick body, which inevitably drew him towards the treatment of consumption. Like Lee, he spent time working as a personal physician on the continent, which gave him the opportunity of adopting the newly invented stethoscope. In the early 1820s, he was in Rome, treating expatriate consumptives, including the poet John Keats. Returning to London in the mid-1820s, he set himself up as a spa physician, visiting resorts during the summer season to treat high-status patients: his therapeutic relationship with Prince Leopold of Belgium may have led to his appointment as a physician in the royal household.^92^

Clark’s success shone a harsh light on Lee’s career. Both were from relatively low-status backgrounds. Both cultivated aristocratic patronage, and Lee was every bit as well connected as Clark. This should have led to a rapid rise through the ranks of the profession, marked by a lucrative private practice. Lee might even have dreamt of a royal position with its accompanying baronetcy. But, unlike Clark, he had been incapable of acquiring the manners essential for professional success. He was aware that his abrasiveness towards fellow professionals had counted against him. Future success could only be achieved by transforming his character: he must attain the virtues of prudence and tact, which he had ‘sacrificed’ through his bad manners. He must use his character to achieve this end.

Initially, Lee acknowledged that jealousy was behind his jaundiced view of Clark. He sought to overcome this by mastering his emotions—giving due credit to Clark for his elevated position. But a view a few days later, his magnanimity failed him when his friend, George Waugh, expressed an ‘unfavourable … opinion’ of Clark’s avarice. ‘He is greedy of fees’. Ultimately, while he might not possess Clark’s tact or prudence, he was at least disinterested.^93^ A few months later, his bad opinion was confirmed when they met in consultation, opening a new avenue to self-improvement:

He is elevated by success – changed in a remarkable manner – avoid him & all such men – Look to something more enduring than Court favour – Strive that your country may not be ashamed of you – I have had many difficulties to struggle with – advantages – early difficulties – an imperfect Scotch education – industry love of my profession.^94^

Lee stressed that overcoming disadvantages could be achieved through diligence and love of the profession. He was fully aware that he shared these disadvantages with Clark, but unlike his ‘elevated’ competitor, he would not be lured by the sophistries of the court. Properly practised, medicine required sacrifice and struggle, but these were no guarantee of reward or success. Lee’s philosophically concluded with a quote: ‘“Banish from your mind all angry feelings of every description.”’^95^

Superficially this was an exhortation to turn away from the envy catalysed by Clark’s success. But the quote itself reveals far more. It was taken from John Gibson Lockhart’s biography of Walter Scott and captures the interaction of life writing with attempted self-fashioning that was crucial to the functioning of a commonplace book. Lockhart’s monumental *Memoirs of the Life of Sir Walter Scott* eventually spanned seven volumes.^96^ Like many biographies of the time, it was a hodgepodge of biographical observation, letters, diaries, personal accounts and more besides. The quote comes from the fifth volume and is a reflection written by the naval officer Basil Hall in the journal he kept while visiting Abbotsford, Scott’s neo-gothic pile near Melrose, for the new year’s celebrations of 1824–25. Hall dwelt upon Scott, his writing practices, anonymous authorship and, above all, his character. He was especially struck by Scott’s ease in the company of others:

How wisely he acts by mixing familiarly with all men, drawing them in crowds around him, placing them at their ease within a near view of his excellence, and taking his chance of being more correctly seen, more thoroughly known, and having his merits more heartily acknowledged, than if, with a hundred times even his abilities, he were to trumpet them forth to the world, and to frighten off spectators to a distance by the brazen sound.^97^

Scott’s natural sympathy made him good company. It was not calculated; it was his sympathetic nature combined with his ability to ‘see through the mists of prejudice and error’, and to find ‘*some* merit in every man, and makes allowances for the faults and weaknesses of all’. The longer passage, however, from which the quote is taken indicates the extent to which Lee used biography to urge the cultivation of self-discipline. What was required most of all was a mastery of the emotions (the clause Lee transcribed is underlined):

by concealing even from himself … every unkindly emotion, he ceases to feel it. His principle is, by every means, to banish from his mind all angry feelings of every description, and then to exempt himself both from the pain of disappointment in disputes where he should fail, and from the pain of causing ill-will in cases where he might succeed. In this way he keeps on good terms with all his neighbours, without exception …. Instead of quarrelling with his eminent brother authors … (as so many others have done, and now do …), he is in friendly and thoroughly unenvious correspondence with them all.^98^

Scott’s ability to manage his emotions and, therefore, his private and professional relations should have provided an object lesson for Lee, and by turning Hall’s observations of Scott into a personal injunction, he demonstrated that he was aware of this. And yet, time and again, he would find himself wanting. He might ‘endeavour … with all his might’, to follow Scott’s example, but he failed to achieve the writer’s emotional mastery. Only a few months later, he gloated at the apparent fall of Clark after the Lady Flora Hastings affair.^99^ Indeed, the garrulous, uncompromising and impetuous character hinted at in the obituaries were fatal flaws in a profession where success was often determined by the manners that enhanced networks and bolstered patronage, and Lee’s journal bears witness to the fact that he was never able to overcome them.

## A Failed ‘Government of the Mind’

Lee attempted to transform his character by banishing untoward emotions through his reading of Scott’s life, but failed. Five years later, in 1843, he was still trying to modify his behaviour when he ‘Resolved to do every thing [*sic*] to make life agreeable & happy by overlooking as far as possible every thing [*sic*], to depress & annoy - & cultivating kind & benevolent feelings’.^100^ Then, in 1852, he was reminding himself that it was ‘obviously’ his ‘duty to prevent extension of error by all means – but it is not necessary to do this in an offensive manner’.^101^ And even in the twilight of his career, he was urging himself to govern his temper ‘by a constant prayer for help to bear the reproaches & various acts of injustice to which I must be exposed … Commit no injustice to others’.^102^ Perhaps his most striking attempt at disciplining himself occurred in 1849 when he conducted a telling thought experiment:

An idea came into mind this morning that I would begin de novo, in London. Set the last 20 years if possible to be forgotten – and act in all respects as if I were just entering London, & had not £30.0.0 and no acquaintance. In fact to make my way a stranger in London:- or supposing all that I have done has been done by an Uncle, who was introduced me with an exhortation to follow his example where it was right & carefully avoid it where wrong. If this idea could be acted upon it would render the remainder of my life short or long, more happy & useful than it would be by following a different course. The habits necessary. Religion Prayer Bible Church. Government of the Mind. Industry. Frugality Temperance in every way.^103^

If he had been able to follow his own advice, he would have prevented the ‘conflict and collision’ that was the inevitable outcome of the toxic mixture of his professional activities and his undisciplined medical self.

But there was an example that appeared to hold out hope; where practice and discovery merged seamlessly, and hard work was rewarded with success. William Hunter, who successfully combined a practice as an accoucheur with a multitude of anatomical discoveries and the foundation of the celebrated Great Windmill Street school, was Lee’s touchstone. When he reflected upon his discovery of the nerves of the uterus, the Hunter brothers were foremost in his mind: ‘If it be as I suppose my fame as an Anatomist placed on a level with the Hunters’.^104^ His diary occasionally reflected upon both brothers, but William was his principal influence, so much so that he was the subject of Lee’s introductory lecture for the 1844–45 session at St George’s medical school and an example of excellence only matched by Harvey in Lee’s Harveian oration.^105^ He was particularly moved when William Hunter Baillie, the son of Matthew Baillie, offered him a lock of William Hunter’s hair.^106^

Lee may well have been struck by parallels to his own life, including his rise from a ‘lowly’ birth, his private practice based upon man-midwifery and the store he set in hard work and diligence. But while he shared a similar practice-setting to Hunter, he was not able to thrive in the same way. Hunter earned £10,000 a year in fees—a figure of which Lee could only dream. Roy Porter identified, through the words of Horace Walpole, the attributes that allowed Hunter to transcend his origins and education. Two of these Lee lacked: the ability to manage the crowd, whether in institutional settings or the public and ‘the techniques of control of self and others’—his diary witness to both these failings.^107^ As much as Lee seemed to will a transformation of his professional self, he was singularly unable to achieve it. Despite using his diary as a tool for disciplining his self, including a deep attention to life writing that frequently had normative functions, he was never able to develop his character in such a way that he could resist ‘conflict and collision’.

## Conclusion: *Bildung*, Character and the Failed Management of the Self

Michael Brown’s *Emotions and Surgery* contains a thought-provoking study of the diary (1812–13) of Henry Oswald that illustrates the unpredictable selves that might emerge from ‘technologies of self’.^108^ The diary testifies to Oswald’s desire to make himself an effective practitioner and yet, according to Brown, he was ‘racked by doubts and anxieties concerning his place in society, his relations with others, and his own state of mind’.^109^ The diary depicts the deep insecurities about professional and social relationships that were often the lot of surgeons and general practitioners. Brown identifies two sources of Oswald’s anxieties: ‘his tenuous social position and the very real risks of disgrace and ruin’; feelings shared by many of his peers.^110^ Whether neophytes, like himself, or older established surgeons, all were vulnerable to the social slights of powerful patrons and the public observation of their risky surgical performances, which, in the pre-anaesthetic era, always teetered on the brink of disaster.

Brown argues that Oswald’s diary was an example of *Bildung*, the German pedagogical theory that emphasised the role of education and knowledge in the cultivation of a fully-rounded self. In Oswald’s diary, *Bildung* was apparent in his ‘private …, attempt to reconcile heart and mind in the formation of the self’.^111^ Brown describes Oswald’s attempts at shaping his behaviour in line with emerging surgical identity, particularly to the romantic ‘emotional regime’ that structured the feelings and actions of his surgical contemporaries. But Oswald’s constant fear was that he would be found wanting. Here, a coherent professional self, shaped in dialogue with a specific social identity, is provisional and always under threat of unravelling. Despite its function as ‘technology of self’, the diary achieves an end that is distinctly unintended, producing a less-than-disciplined self.

Other studies of individual self-formation through the use of ‘technologies of self’ present different outcomes with culture, identity and science seemingly coming together to create fully-formed and coherent professional selves. Ian Hesketh’s analysis of the physicist John Tyndall’s self-fashioning through his journal is an example of this.^112^ Tyndall’s scientific self was forged through the interaction of his diary with the epistemic regime of ‘mechanical objectivity’, which, according to Lorraine Daston and Peter Galison, was embedded in the identity of mid-nineteenth-century science.^113^ The scientist’s strength of character was measured by the extent to which the will could be disciplined to accept the often counter-intuitive evidence provided by mechanical tools of observation (for example, time-lapse photography), while ignoring the subjective desires of the potentially unruly will. Tyndall used his journal to fashion his scientific self along these lines. The journal documented his constant attention to stoic and spartan practices that would ensure his unwavering commitment to ‘mechanical objectivity’. In transcendental philosophy, he found a resource that buttressed his efforts to achieve this will-less science; the journal enforcing and reinforcing rigorous mental and bodily discipline and the denial of pleasure that was essential to this mode of scientific practice. It was a constant struggle, but working through his ‘technology of self’, Tyndall successfully produced the scientific self that was his end-goal.

Like Oswald and Tyndall, Lee was engaged in a project of self-cultivation. While the short time-span of Oswald’s diary limits its usefulness as a point of comparison, it does indicate how medical men worked towards a specific identity, while his struggles resonate with those of Lee in various ways, not least the shared desire to be recognised as respected and respectable practitioners. Both were aware, too, of their vulnerability and that, like Sir James Clark, they were just one bad operative performance or one misdiagnosis away from disgrace. Tyndall’s journal, on the other hand, bares striking similarities to Lee’s. Both were engaged in reading to cultivate their professional selves. Both found force in particular genres (philosophy for Tyndall, biography for Lee) that directed the individual towards an ideal embedded in specific professional identities (‘mechanical objectivity’ for Tyndall, practitioner and anatomical discoverer for Lee). These identities demanded adherence to a set of identity-based norms that valued a range of virtues from collegiality to aestheticism. Most importantly, regardless of the identity regime to which each man adhered, there was a single mechanism central to their self-fashioning: character.^114^

Character, it was believed, was critical to directing the will towards acceptable moral goals. A badly formed character not only led to an undisciplined self but also made it visible to peers. Lee, himself, was acutely aware of the importance of bearing a good professional character, hence his focus upon biographical subjects who illustrated its good and bad effects. Indeed, beyond the context of the case studies presented in this article, ‘character’ cropped up many times as an exhortation to moral transformation or as a critique of his peers’ shortcomings.^115^ At the same time, we have witnessed Lee’s inability to transform his own character despite his diary as a ‘technology of self’. Most often this took the form of a failure of emotional management. Many of the lives he raised in his diary provided examples from which he could have and should have learned. Ferguson’s Bell, for example, counselled him towards a proper cultivation of professional manners, while warning him that his discoveries might not be justly rewarded. Brodie’s *Autobiography* triggered an uncontrollable rage that the earlier example of Walter Scott could not rectify. William Hunter should have been a beacon for self-management but was not. Despite each life presenting a moral, none altered his character and his self remained resolutely unprofessional.

Finally, I would like to draw a series of wider conclusions relating to character, medical identity and technologies of self. Firstly, a social identity (including professional identity) can be usefully thought of as moral framework providing an *ethos* for shaping the selves of individuals. This shifts our perspective from describing the attributes of an identity to the virtues integral to that identity. Social identity is always ethical. Whether laying down appropriate knowledge or practice, it instructs the individual about who they are, what they should be and how they should behave.^116^ Secondly, character opens up a space to think more deeply about how identities are performed. Building upon the work of John Harley Warner and Michael Brown, by identifying what exactly needs to be policed, it provides insights into the making and enforcing of boundaries.^117^ Thirdly, it cautions us about accepting representations of identity at face value. In other words, it shows that specific identities were not simply imposed upon the self; character reveals the intricate mediation between identity and self, where the resulting and dynamic self is never a perfect mirror of available identities—‘appearance is not being’, as one critic of Daston and Galison’s *Objectivity* summarised the collapse of representation into reality that is implied by this and similar theoretical perspectives.^118^

In the final analysis, there is an undeniable tension sitting at the heart of Lee’s self-reflections. Caught between the competing norms of the emerging medical profession, which emphasised both the field of correct behaviour and the rewards due to a successful discoverer, he appeared to believe that if only he could transform his character to coincide with those norms, the obstacles to professional success, in all its guises, would crumble. This certainly appeared to be the message conveyed to him by his biographical reading. But he failed to impose those norms upon his self and thus revealed a flaw in his character. Unfortunately for Lee, his behaviour—read as the outward and publicly available symptoms of his inner self—indicated the true nature of his character, both to himself and his enemies. Ironically, in Lee’s case, ‘appearance’ could be read as ‘being’, although the being in question did not align with emergent professional identities.

Thus, Lee’s diary shows that at the levels of ethics and performance, his character was found wanting, and he failed to ‘attain’ that ‘certain state of perfection, happiness, purity, supernatural power’ that was the end-goal of a Foucauldian ‘technology of self’. Returning to Lee’s abstract of Bell’s obituary which he transcribed over 30 years before his own death, one passage reads not so much as a warning about bad character but as an uncanny verdict on his own:

how easy to say why they [Bell’s contemporaries] did not succeed in the game of life – the manner, the propensity, a passion, pride, jealousy – bad temper have reduced many who might have risen if measured by their abilities & arguments. Yet how difficult to change that one trait on which all depends.^119^

Lee might well have recognised all of these failings in his own conduct. He had, however, been singularly unsuccessful in his efforts to change ‘that one trait’.

## Footnotes

1 Anon., ‘Royal Medical and Chirurgical Society’, *BMJ*, 1877, 1, 305–307. Anon., ‘Royal Medical and Chirurgical Society’, *The Lancet*, 1877, 1, 347–49; Anon., ‘Royal Medical and Chirurgical Society’, *The Medical Times and Gazette*, 1877, 1, 273–74. Further references to the speech will refer to the *BMJ*.

2 Anon., ‘Royal Medical and Chirurgical Society’, *BMJ*, 306.

3
*Ibid*.

4
*Ibid*., 307.

5 For an overview of Lee’s life see Hilary Marland, ‘Lee, Robert (1793–1877), Physician Specializing in Gynaecology and Obstetrics’, in *Oxford Dictionary of National Biography* (Oxford: Oxford University Press, 2004), https://doi.org/10.1093/ref:odnb/16306.

6 Adrian Desmond, *The Politics of Evolution: Morphology, Medicine and Reform in Radical London* (Chicago: University of Chicago Press, 1992), 393–95.

7 Anon., ‘Biographical Sketch of Robert Lee, M.D., F. R. S’, *Lancet*, 1851, 1, 332–37.

8 Ann Dally, *Women Under the Knife: A History of Surgery* (London: Hutchinson Radius, 1991), 127, 138–40; Sally Frampton, *Belly-Rippers, Surgical Innovation and the Ovariotomy Controversy* (Basingstoke: Palgrave Macmillan, 2018), 74; Ornella Moscucci, *The Science of Woman: Gynaecology and Gender in England, 1800-1929* (Cambridge: Cambridge University Press, 1990), 113–15, 137–40, 152–53; Mary Poovey, ‘“Scenes of an Indelicate Character”: The Medical “Treatment” of Victorian Women’, *Representations*, 1986, 14, 137–68, 154.

9 For ovariotomy, Frampton, *Belly-Rippers*, 75–78, examines Lee’s mobilisation of statistics to demolish antagonists like Frederick Bird.

10
*Ibid.*, 2.

11 Anon., ‘Biographical Sketch’, 337.

12
*Transactions of the Obstetrical Society of London*, Vol. 1–20, shows that he was never a Fellow of the Society which was founded in 1858.

13 Anon., ‘Dr Robert Lee’, *BMJ*, 1877, 1, 177.

14 Anon., ‘Annotations’, *The Lancet*, 1877, 1, 215.

15 Anon., ‘Robert Lee, M.D., F.R.C.P., F.R.S.’, *The Medical Times and Gazette*, 1877, 1, 217–18.

16 Michael Brown, *Performing Medicine: Medical Culture and Identity in Provincial England, c. 1760-1850* (Manchester: Manchester University Press, 2011), 159, uses Benedict Anderson’s ‘imagined communities’ to discuss the role of medical journals in making identity; Sally Frampton, ‘“A Borderland in Ethics”; Medical Journals, the Public, and the Medical Profession in Nineteenth-century Britain’, in Gowan Dawson *et al*., eds, *Science Periodicals in Nineteenth-Century Britain: Constructing Scientific Communities* (Chicago: University of Chicago Press, 2020), 311–36 explores journals policing boundaries in the late nineteenth century.

17 Wellcome Archives, Lee, Robert (1793–1877) and Lee, Robert James (1841–1924), MSS.3213–26 and 5469. These papers were a bequest from the pathologist Norah Schuster. Why Schuster possessed them is hard to fathom. The papers themselves had been preserved by Lee’s son, Robert James, for writing his father’s biography.

18 Diary of Robert Lee, 1837–77 (henceforth ‘Diary’), Wellcome Library, MS 3218. Because the folio numbers are inconsistent, all references are to date of entry. The last entry was 19 April 1873.

19 See for example, William Sharpey to James Young Simpson, letter dated 23 Oct. [catalogued 1856; context suggests 1845–46], Archives of the Royal College of Surgeons Edinburgh, JYS 10/7/28. The Simpson papers (e.g. JYS 1/2/65, 1/3/4, 2/2/1, 2/2/2-3, 2/2/59, 5/1/1, 8/1/5/2) indicate the depth of a mutual hatred that transcended the fault-lines between the conservative and innovative approaches underwriting their controversies.

20 Michel Foucault, *Ethics: Subjectivity and Truth*, ed. Paul Rabinow (New York: New Press, 1997), ‘Sexuality and Solitude’, 175–84; ‘Self Writing’, 207–22 and ‘Technologies of the Self’, 223–52.

21 Foucault, ‘Sexuality’, 177.

22 Stefan Collini, ‘The Idea of “Character” in Victorian Political Thought’, *Transactions of the Royal Historical Society*, 1985, 35, 29–50.

23 Pierre Bourdieu’s concept of habitus is used by Brown, *Emotions and Surgery*, 22 and 33. Notoriously difficult to read, the easiest gateway to Bourdieu’s habitus is ‘Sport and Social Class’, *Social Science Information*, 1978, 17, 819–40.

24 Character in its literary and descriptive senses dominates Brown’s *Performing Medicine*, although his exploration of medico-gentility, 48–81, explores evaluative ‘character’; Alison Moulds, *Medical Identities and Print Culture, 1830s-1910s* (Cham: Palgrave Macmillan, 2021) mainly uses ‘character’ in its literary or descriptive senses; Michael Brown, *Emotions and Surgery in Britain, 1793-1912* (Cambridge: Cambridge University Press, 2022), gets close to exploring character as a mechanism for controlling the will—in particular, in his analysis of Oswald’s diary, 71–76 (see below).

25 Samuel Smiles, *Self-Help; With Illustrations of Character and Conduct* (London: John Murray, 1859), distilled many traits of character found in Lee’s diary.

26 Brown, *Performing Medicine* and Moulds, *Medical Identities*.

27 Jan Golinski, *The Experimental Self: Humphry Davy and the Making of a Man of Science* (Chicago: University of Chicago Press, 2016).

28 Andrew Cunningham, ‘The Pen and the Sword: Recovering the Disciplinary Identity of Physiology and Anatomy before 1800: I: Old Physiology—the Pen’, *Studies in History and Philosophy of Science Part C: Studies in History and Philosophy of Biological and Biomedical Sciences*, 2002, 33, 631–65, and ‘II. Old Anatomy the Sword’, *Studies in HPS. Part C*, 2003, 34, 51–76.

29 Robert Lee, ‘Observations on the Discovery of the Original Obstetric Instruments of the Chamberlens’, *Medico-Chirurgical Transactions*, 1862, 27, 1–24.

30 Stana Nenadic, ‘Writing Medical Lives, Creating Posthumous Reputations: Dr Matthew Baillie and His Family in the Nineteenth Century’, *Social History of Medicine*, 2010, 23, 509–27, 518.

31 John Tosh, ‘Masculinities in an Industrializing Society: Britain, 1800-1914’, *Journal of British Studies*, 2005, 44, 330–42.

32 Anon., *Literary Diary; Or, Improved Common-Place-Book on the Plan Recommended by Mr. Locke* (London: Taylor and Walton, n.d.).

33 David Allan, *Commonplace Books and Reading in Georgian England* (Cambridge University Press, 2010); Jillian M. Hess, *How Romantics and Victorians Organized Information: Commonplace Books, Scrapbooks, and Albums* (Oxford: Oxford University Press, 2022).

34 Anon., *Literary Diary*, 3.

35 Ken Autrey, ‘Toward a Rhetoric of Journal Writing’, *Rhetoric Review*, 1991, 10, 74–90.

36 See Rebecca Steinitz, *Time, Space, and Gender in the Nineteenth-Century British Diary* (New York: Palgrave Macmillan, 2011); Philippe Lejeune, *On Diary* (Honolulu: University of Hawaii Press, 2009); Peter Heehs, *Writing the Self: Diaries, Memoirs, and the History of the Self* (New York: Bloomsbury Academic, 2013).

37 Ella Ophir, ‘The Diary and the Commonplace Book: Self-Inscription in “the Note Books of a Woman Alone”’, *Biography*, 2015, 38, 41–55, discusses the overlap between diaries and commonplace books.

38 Nenadic, ‘Writing Medical Lives’; David Amigoni, *Victorian Biography: Intellectuals and the Ordering of Discourse* (Abingdon: Routledge, 1993).

39
*Ibid.*, 514.

40 M. Jeanne Peterson, *The Medical Profession in Mid-Victorian London* (Berkeley: University of California Press, 1978), 172–3; Anne Digby, *Making a Medical Living: Doctors and Patients in the English Market for Medicine, 1720-1911* (Cambridge: Cambridge University Press, 1994), 174.

41 For example, see the brief descriptions below about his disputes with James Young Simpson over *placenta praevia* and Robert Paterson over the *corpus luteum*.

42 Diana E. Manuel, ‘Robert Lee, the Uterine Nervous System and a Wrangle at the Royal Society, 1839-1849’, *Journal of the Royal Society of Medicine*, 2001, 94, 645–47.

43 Robert Lee, *Memoirs on the Ganglia and Nerves of the Uterus* (London: J. Churchill, 1849), 12; Letter from Astley Cooper to Lee, ‘Diary’ dated 17 August 1840.

44 Robert Lee, ‘On the Nervous Ganglia of the Uterus’, *Philosophical Transactions of the Royal Society of London*, 1841, 131, 269–75.

45 Manuel, ‘Robert Lee’, 647; Desmond, *Politics of Evolution*, 393–94.

46 Robert Knox, ‘Nerves of the Uterus.—Controversy Between Dr Lee and the Council of the Royal Society’, *Medical Times*, 1846, 14, 387–88, 388.

47 Anon. ‘The Royal Society. Report of the Physiological Committee of the Royal Society on Dr Robert Lee’s Paper, entitled “On the Ganglia and Nerves of the Heart”’, *London Medical Gazette*, 1848, 6, 82–84; later that year, in a paper read at the Royal Medico-Chirurgical Society, Lee reported about the nerves of the heart (to the chagrin of the Society’s president), ‘These nerves have been manufactured by nature, and not by him (Dr Lee), as has been alleged by some’, 1088–89.

48 Diary, 15 December 1838, short obituary of John Hunter’s son, source not found; 13 February 1841, Astley Cooper, from *Morning Chronicle*, 13 February 1841; 19 April 1841, comments on the life of Mahmud, probably taken from Mountstuart Elphinstone, *The History of India*, 2 vols (London: John Murray, 1841), 1, 570; 15 November 1841, ‘Reading the life of Lord Clive’; Justice Story, American Jurist, no date, but extracted from the *Morning Chronicle*’s obituary, 13 October 1845, 6; No date, but on or before 26 December 1845, Baron Guillaume Dupuytren, who died in 1835, abstracted from Anon. ‘Memoir of Dupuytren’, *Lancet*, 1834, 1, 820–25; 11 July 1846, Lord Chief Justice Sir Nicolas Conyngham Tindal, quote from Anon., ‘Death of Lord Chief Justice Tindal’, *The Times*, 8 July 1846, 5, but quote also reproduced in the obituary in *The Spectator*, 11 July 1846, 656; Thomas Chalmers counterposed with quote about the Rev. George Whitefield, no date but after entry for 10 July 1847, Chalmers’s obituary from, Anon., ‘Recollections of Dr Chalmers’, *Fraser’s Magazine*, 1847, 36, 140–55, quote on Whitefield could have been taken from any number of sources, but most likely Robert Philip, *The Life and Times of the Reverend George Whitefield, M.A.* (London: George Virtue, 1838), 556; ‘Reading the memoirs of Dr Priestly [*sic*]’, no date but around Spring 1848, directly quotes *Memoirs of Dr Joseph Priestley to the Year 1795* (London: J. Johnson 1806), 110, but could also found several other publications including John Towill Rutt, *Life and Correspondence of Joseph Priestley*, 2 vols (London: R. Hunter, M. Eaton & C. Fox, 1831), 1, 347.

49 Diary, March (no day) 1841.

50 Diary, 19 March 1841.

51 Donna T. Andrew, ‘The Suicide of Sir Samuel Romilly: Apotheosis or Outrage?’, in Jeffrey Watt, ed., *From Sin to Insanity: Suicide in Early Modern Europe* (Ithaca: Cornell University Press, 2019), 175–88.

52 Diary, 19 April 1841.

53 Anon., *The Morning Chronicle*, 21 July 1838, 3.

54 Diary, no date (entry just before 23 July 1838).

55 Diary, 23 July 1838.

56
*Ibid.*

57 See, for example, Diary, 8 December 1838 on Mark Brown; 5 March 1839, on James Clark; 27 June 1840 on Samuel Dr Ashwell.

58 Diary, 1 January 1844.

59 Carin Berkowitz, *Charles Bell and the Anatomy of Reform* (Chicago: University of Chicago Press, 2015), 4.

60 Diary, no date (‘summer’) 1843, abstracting Anon. [Robert Ferguson], ‘Article VII’ [review of selected works by Bell] *Quarterly Review*, 1843, 72, 192–231; around or after middle of July 1843, abstracting Anon., ‘The Rival Discoverers’, *London Medical Gazette*, 1837-1838, 1, 903–6; no date but immediately after the entry ‘On the 1 Septr 1846 I entered on my 53 year’, suggesting that during September, he had abstracted Anon., ‘Resistance to Great Truths. Bell and the Nervous System’, *Chambers’s Edinburgh Journal*, 26 September 1846, 206–7.

61 Anon., ‘Article VII.’, 192.

62 Lee identified Ferguson as the author, confirmed by the *Wellesley Index*; Anon., *Letters of Sir Charles Bell* (London: John Murray, 1870).

63 Letter pasted into the diary, dated 27 March 1867, from Alexander Shaw to Robert Lee.

64 Amedée Pichot, *The Life and Labours of Sir Charles Bell* (London: Richard Bentley, 1860), drew heavily on Ferguson’s obituary.

65 Copied from Anon., ‘Article VII.’, 227.

66
*Ibid.*, 228.

67
*Ibid.*, 225-26.

68 Lee moved his extended family to Brighton for several reasons, including the health of his wife and mother-in-law, and his sons’ attendance at Brighton College.

69 Diary, after 5 July 1843 quoting Anon., ‘The Rival Discoverers’, 903.

70
*Ibid.*

71
*Ibid.*

72
*Ibid.*

73 Desmond, *Politics of Evolution*, 231–33.

74 Diary, undated, retrospective entry made some time after January 1845.

75 Anon., ‘Resistance to Great Truths’, 206–7.

76 Diary, after 11 August 1846.

77 Anon., ‘Resistance to Great Truths’, 207.

78 Diary, 16 April 1864.

79 Benjamin Brodie, *Autobiography of the Late Sir Benjamin C. Brodie, Bart* (London: Longmans, Green, and Co., 1865).

80 See Diary, entry after 24 April 1845 (probably made around 1849); January and February 1861 and 20 March 1861.

81 Diary, 19 March 1865.

82 Diary, around 28 March 1865.

83 Diary, 11 April 1866.

84 Diary, 10 September 1866.

85 Wellcome Archives, Correspondence and Papers by Robert Lee and Robert James Lee, MS.5469.

86 James Young Simpson, ‘Some Remarks on the Treatment of Unavoidable Haemorrhage by Extraction of the Placenta Before the Child. With a Few Observations on Dr Lee’s Practice’, *London Medical Gazette*, 1845-1846, 1, 1009–16. The *Gazette* published the personal correspondence between them in ‘Supplement’, 1083–90. Lee’s formal response in the same volume was ‘Objections to the Practice of Tearing Away the Placenta, Instead of Turning the Child, in Cases of Unavoidable Uterine Haemorrhage’, 1106–10.

87 Anon., ‘Controversy Between Dr Simpson and Dr Lee’, *Medical Times*, 1845–1846, 13, 140–41, 141.

88 Robert Lee, ‘Lectures on the Theory and Practice of Midwifery, Delivered in the Theatre of St. George’s Hospital. Lecture V. On the Anatomy and Physiology of the Gravid Uterus’, *London Medical Gazette*, 1842–1843, 1, 193–99, 198–99; Robert Paterson, ‘Answer to Some Criticisms of Dr Lee on The Character of Early Corpora Lutea’, *London Medical Gazette*, 1842–1843, 1, 365–68.

89 Diary, 14 July 1838.

90 Head of Diary page covering 10–14 July.

91 Diary, 10 July 1838.

92 R. A. L. Agnew, ‘Clark, Sir James, First Baronet (1788–1870), Physician’, in *Oxford Dictionary of National Biography* (Oxford: Oxford University Press, 2019), https://doi.org/10.1093/ref:odnb/5463.

93 Diary, 14 July 1838.

94 Diary, 9 September 1838.

95 Diary, 10 July 1838.

96 John Gibson Lockhart, *Memoirs of the Life of Sir Walter Scott, Bart*, 7 vols (Edinburgh & London: Robert Cadell, John Murray & Whittaker & Co., 1837–1838).

97 Lockhart, *Memoirs of The Life of Sir Walter Scott*, *5*, 416.

98
*Ibid.*, 417.

99 Diary, 5 March 1839. Subject of much satire, Clark had misdiagnosed the tumour of Lady Florence Hastings as pregnancy. Clark lived to fall again, particularly with his misdiagnosis of Prince Albert during his final illness.

100 Diary, 15 June 1843.

101 Diary, 23 November 1852.

102 Diary, 1 January 1864.

103 Diary, 5 April 1849.

104 Diary, entry after 6 January 1840.

105 Robert Lee, ‘The Life and Labours of Dr William Hunter’, *London Medical Gazette*, 1844–1845, 1, 1–11; Royal College of Physicians London, ‘The Harveian Oration Delivered at the Royal College of Physicians on June 26 1864 by Robert Lee MA FRS’, MS.1024/411 f.32 (Latin version), English translation has no folio numbers.

106 Diary, 9 June 1861.

107 Roy Porter, ‘William Hunter: A Surgeon and a Gentleman’, in W. F. Bynum and Roy Porter, eds, *William Hunter and the Eighteenth-Century Medical World* (Cambridge: Cambridge University Press, 1985), 7–34, 34.

108 Brown, *Emotions and Surgery*, 71–76.

109
*Ibid*., 73.

110
*Ibid*., 74.

111
*Ibid.*, 75.

112 Ian Hesketh, ‘Technologies of the Scientific Self: John Tyndall and his Journal’, *Isis*, 2019, 110, 460–82.

113 Lorraine Daston and Peter Galison, *Objectivity* (Cambridge, Mass.: Zone Books, 2007).

114 Hesketh, ‘Technologies of the Scientific Self’, 468–69.

115 See, for example: Diary: 23 August 1838; 15 December 1838; 5 March 1839; 28 April 1839; 1–2 July 1839; 19 March 1841; 2 February 1855; 15 July 1856.

116 Here I am particularly influenced by Anthony Appiah, *The Ethics of Identity* (Princeton, NJ: Princeton University Press, 2005); Ian Hacking, ‘Kinds of People: Moving Targets’, *Proceedings of the British Academy*, 2007, 151, 285–317; Charles Taylor, *Sources of the Self: The Making of the Modern Identity* (Cambridge, Mass: Harvard University Press, 1989).

117 John Harley Warner, *The Therapeutic Perspective: Medical Practice, Knowledge, and Identity in America, 1820-1885* (Cambridge, Mass: Harvard University Press, 1986); Brown, *Performing Medicine*.

118 Esha Shah, *Who Is the Scientist-Subject? Affective History of the Gene* (London: Routledge, 2018), 30–32.

119 Diary, no date (‘summer’) 1843, copied from Anon., ‘Article VII.’, 227.

## Notes

James Bradley is a senior lecturer in the History and Philosophy of Science at the University of Melbourne. He is currently engaged in a project exploring virtue, ethics and identity in the Victorian medical profession. However, this article is part of a wider biographical project about Robert Lee provisionally titled ‘Robert Lee and his enemies’.

